# Long noncoding RNA small nucleolar RNA host genes as prognostic molecular biomarkers in hepatocellular carcinoma: A meta‐analysis

**DOI:** 10.1002/cam4.7200

**Published:** 2024-04-17

**Authors:** Meng Huang, Zhiwen Zhao, Lihua Yang

**Affiliations:** ^1^ Medical Center for Digestive Disease The Second Affiliated Hospital of Nanjing Medical University Nanjing Jiangsu Province China

**Keywords:** Clinicopathological features, hepatocellular carcinoma, meta‐analysis, prognosis, SNHGs

## Abstract

**Background:**

Recently, increasing data have suggested that the lncRNA small nucleolar RNA host genes (SNHGs) were aberrantly expressed in hepatocellular carcinoma (HCC), but the association between the prognosis of HCC and their expression remained unclear. The purpose of this meta‐analysis was to determine the prognostic significance of lncRNA SNHGs in HCC.

**Methods:**

We systematically searched Embase, Web of Science, PubMed, and Cochrane Library for eligible articles published up to February 2024. The prognostic significance of SNHGs in HCC was evaluated by hazard ratios (HRs) and 95% confidence intervals (CIs). Odds ratios (ORs) were used to assess the clinicopathological features of SNHGs.

**Results:**

This analysis comprised a total of 25 studies covering 2314 patients with HCC. The findings demonstrated that over‐expressed SNHGs were associated with larger tumor size, multiple tumor numbers, poor histologic grade, earlier lymphatic metastasis, vein invasion, advanced tumor stage, portal vein tumor thrombosis (PVTT), and higher alpha‐fetoprotein (AFP) level, but not with hepatitis B virus (HBV) infection, and cirrhosis. In terms of prognosis, patients with higher SNHG expression were more likely to have shorter overall survival (OS), relapse‐free survival (RFS), and disease‐free survival (DFS).

**Conclusions:**

In conclusion, upregulation of SNHGs expression correlates with shorter OS, RFS, DFS, tumor size and numbers, histologic grade, lymphatic metastasis, vein invasion, tumor stage, PVTT, and AFP level, suggesting that SNHGs may serve as prognostic biomarkers in HCC.

## 1 INTRODUCTION

According to 2020 Cancer Data, hepatocellular carcinoma (HCC) is the sixth most prevalent cancer and the third leading cause of cancer death worldwide, resulting in a significant disease burden.^1^ Metabolic risk factors for HCC are becoming more prevalent despite the hepatitis virus remaining the main cause.^2^ At the moment, nondrug treatments include ablation, transarterial chemoembolization (TACE), liver transplantation, and hepatic resection. In the meanwhile, advanced HCC is primarily treated systemically with medications including monoclonal antibodies like nivolumab and small molecule targeted medications like sorafenib and lenvatinib.^3^ Most patients are diagnosed in the middle or late stages, with a 5‐year survival rate below 18%.^4^ However, current therapy advances still fail to provide satisfactory outcomes. Therefore, it is critical and vital to develop effective biomarkers early on that can be applied as targeted therapy for HCC.

Long noncoding RNAs (LncRNAs) are noncoding RNAs with a length greater than 200 nucleotides. In the absence of functional open reading frames, they hardly encode any protein.^5^ Increasing studies have clarified that lncRNAs, initially considered as genomic junk, can act as oncogenes or antioncogenes in cancer, regulating tumor growth, metastasis, metabolism, and progression, and may offer a new method for cancer diagnosis and treatment.^6^ Multiple lncRNAs about HCC have been found to exhibit abnormal expression and take a role in malignant phenotypes via binding to DNA, RNA, or proteins or by encoding tiny peptides, which could influence the progression of HCC. They were expected to be potential biomarkers for diagnosis and prognosis of HCC.^7^

Long noncoding small nucleolar RNA host genes (lnc‐SNHGs) are host genes for snoRNAs (small nucleolar RNAs), which are overexpressed in human cancers. When acting as a lncRNA called SNHG, snoRNAs retain full‐length transcripts, including exons.^8^ Five different molecular mechanisms explain how SNHGs work: three are connected to cytoplasmic localization, reducing miRNA bioavailability through molecular sponge activity, limiting translation, and preventing ubiquitination; the other two are related to nuclear localization, interacting with transcription factors, or repressors and altering DNA methylation. SNHGs are crucial in carcinogenesis and cancer progression through influencing DNA, RNA, and protein.^9^ Additionally, SNHGs take part in the occurrence, growth, and pathophysiology of gastrointestinal cancers by regulating downstream targets. They also correlate with clinicopathological traits such as shorter overall survival (OS), tumor size, lymph node metastasis, and TNM stage.^10^ In recent years, studies have shown that multiple SNHGs was significantly upregulated in HCC and was strongly associated with clinicopathological features.^11^

According to an examination of experimental data, HCC patients expressing high levels of SNHGs got a worse prognosis. Various studies have confirmed their predictive value, although it is unclear how each of them affects the prognosis of HCC.^12^ In 2017, Li et al.^13^ published a meta‐analysis on SNHGs and HCC prognosis, but there were only five SNHGs included. In recent years, studies involving more SNHGs about HCC have emerged. So for further knowledge, we carried out a meta‐analysis on the prognostic value and clinicopathological features of the SNHG family in HCC.

## 2 METHODS

### 2.1 Literature searching strategies

We prospectively registered the meta‐analysis on PROSPERO (CRD42022370591). To filter all pertinent studies up to February 2024, Embase, Web of Science, PubMed, and Cochrane Library were searched online. The following keywords were used in the search: (SNHG1 OR SNHG2 OR SNHG3 OR SNHG4 OR SNHG5 OR SNHG6 OR SNHG7 OR SNHG8 OR SNHG9 OR SNHG10 OR SNHG11 OR SNHG12 OR SNHG13 OR SNHG14 OR SNHG15 OR SNHG16 OR SNHG17 OR SNHG18 OR SNHG19 OR SNHG20 OR SNHG21 OR SNHG22) AND ((HCC) OR (Liver Cancer) OR (Liver Cell Carcinoma) OR (Hepatoma)) AND (prognosis OR prognostic OR outcome). The specific searching strategies in each database were provided in the Table S1. To make sure that all appropriate studies were included, we also manually searched pertinent original article references. Two researchers independently decided which publications should be included and excluded. Disagreements were resolved by consensus after discussion with the third author.

### 2.2 Inclusion and exclusion criteria

#### 2.2.1 Inclusion criteria

(A) Patients were definitely diagnosed as HCC, and their SNHG expression was detected by specific methods, (B) “High SNHG” and “low SNHG” groups were created. (C) The correlation between SNHG and prognosis along with clinical features were reported, (D) there was enough data in the studies to generate hazard ratios (HRs) and 95% confidence intervals (CIs), (E) studies were published in English.

#### 2.2.2 Exclusion criteria

(A) Replicated studies, (B) review, meta, raw letter, cellular, and animal studies, (C) irrelevant topics, (D) inadequate information for calculation of HRs and 95% CIs.

### 2.3 Data extraction and quality assessment

Data were separately extracted by two researchers using inclusion and exclusion criteria, and any differences were solved by discussion. We extracted the following information: the first author, study country, publication year, sample size, sample type, SNHG type, method of SNHG detection, the cutoff value of SNHG expression, endpoints, extract method of HR, HRs and 95% CIs of OS/relapse‐free survival (RFS) /disease‐free survival (DFS) reported or estimated from survival curves, and clinical characteristics including age, sex, lymphatic metastasis, vein invasion, tumor size, tumor stage, histologic grade. If only Kaplan–Meier curves were given, we could calculate HR and 95% CI with the Engage Digitizer v11.1 software based on the strategy suggested by Tierney.^14^

The Newcastle‐Ottawa Quality Assessment Scale (NOS) was employed to examine the quality of the included studies. The NOS scale^15^ considers inclusion, outcome, and comparability, with scores from 0 to 9. Studies with a score of 6 or higher were included in the meta‐analysis.

### 2.4 Statistical analysis

The STATA 15.0 software was used to conduct this meta‐analysis of all eligible studies. The relationship between SNHG expression and the prognosis of HCC patients was evaluated by HRs and 95% CIs. Additionally, odds ratios (ORs) and 95% CIs were used to analyze the association between clinicopathological traits and SNHG. We analyzed heterogeneity between studies with the Chi‐square *Q* test and I^2^ statistic. The random‐effects model was applied if there was significant heterogeneity (*χ* ^2^ test *p* <0.1 or I^2^ >50%); otherwise, a fixed‐effects model was utilized. A sensitivity analysis was conducted to check whether the findings were stable. Begg's and Egger's tests were used to assess publication bias. Significant differences were defined as *p*‐values <0.05.

## 3 RESULTS

### 3.1 Search process and features of included literature

**FIGURE 1:**
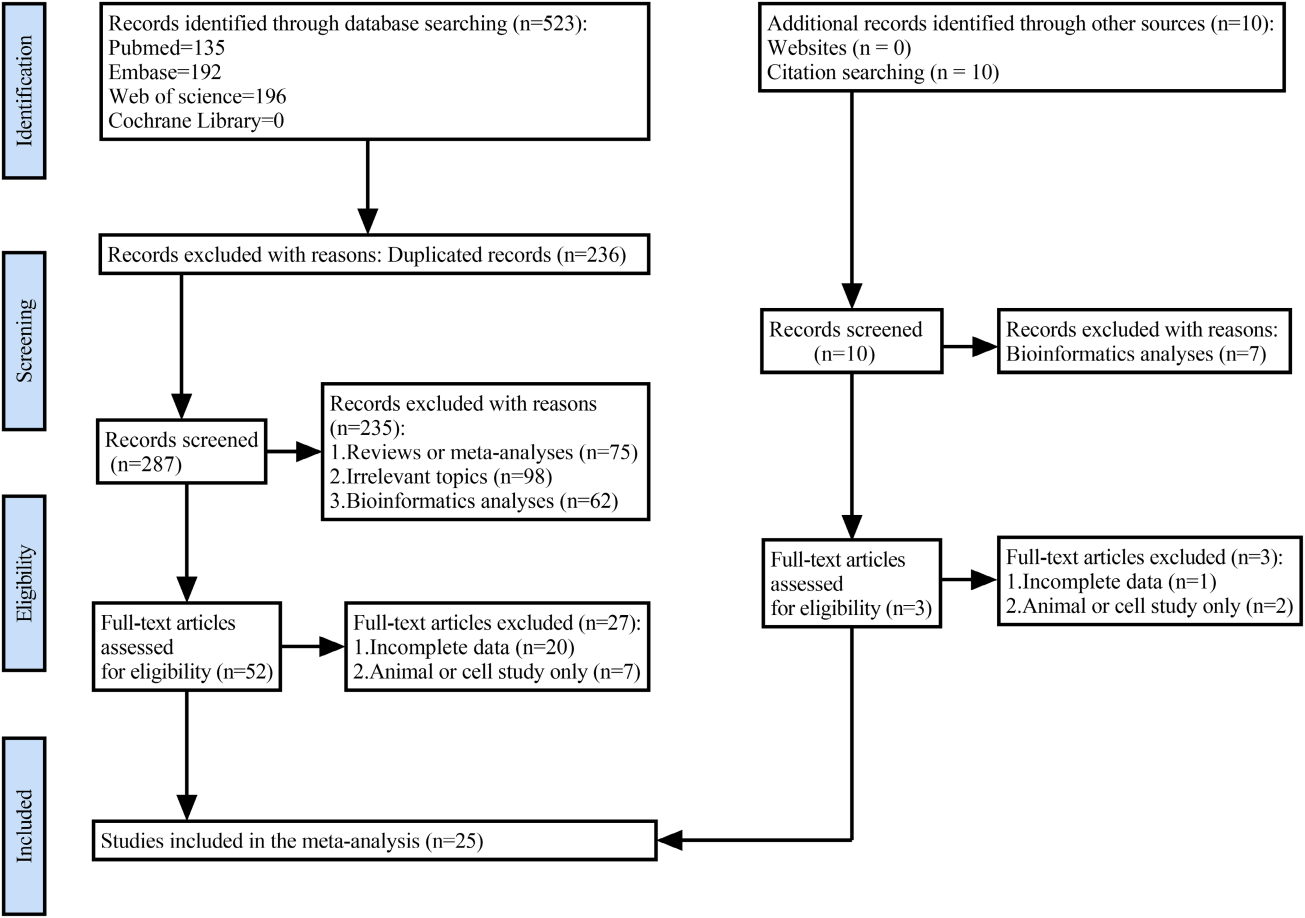
PRISMA flowchart of the selection process.

The detailed search and inclusion process is shown in Figure 1. There were 533 pieces of literature found overall in the initial search. We chose 55 studies strictly based on the criteria for inclusion and exclusion. Then, we further excluded 30 studies upon reviewing all full text of the remaining 55 articles. Ultimately, this meta‐analysis included 25 publications (2314 patients).^16^, ^17^, ^18^, ^19^, ^20^, ^21^, ^22^, ^23^, ^24^, ^25^, ^26^, ^27^, ^28^, ^29^, ^30^, ^31^, ^32^, ^33^, ^34^, ^35^, ^36^, ^37^, ^38^, ^39^, ^40^ The sample size was between 40 and 160, with publication years ranging from 2016 to 2022. Except for one,^38^ all of the listed studies were carried out in China. These eligible studies included 2314 patients with 13 types of SNHGs, involving SNHG1,^19^, ^35^ SNHG3,^20^, ^29^ SNHG6,^16^, ^21^ SNHG7,^27^, ^28^, ^31^, ^32^ SNHG9,^38^ SNHG10,^25^ SNHG11,^30^ SNHG12,^22^ SNHG15,^18^, ^34^ SNHG16,^24^, ^26^, ^33^, ^40^ SNHG17,^37^ SNHG18,^39^ SNHG20,^17^, ^23^ and SNHG22.^36^ Based on SNHG expression, the enrolled patients were divided into the “high SNHG” or “low SNHG” group. Apart from one study that identified SNHG expression in venous blood,^38^ all of the research detected SNHG expression in tissue by qRT‐PCR or ISH. The correlation between SNHG and OS was explored in every study. Of them, eight studies also reported the correlation between SNHG and DFS/RFS. Thirteen studies gave HR values directly, while Kaplan–Meier curves were available for calculating HR values in the remaining studies. All included studies had NOS scores ≥6. The characteristics of the included literature are detailed in Table 1.

**TABLE 1:** Details of included studies.

Author	Year	Country	SNHG type	Sample size	Sample	Outcome	Detection method	Cutoff value	Follow‐up (months)	Extract method of HR	NOS
Chang, L	2016	China	SNHG 6	80	Tissue	OS, RFS	qRT‐PCR	Median	36	Reported	8
Zhang, D	2016	China	SNHG 20	144	Tissue	OS,DFS	ISH	3	60	Reported	9
Zhang, JH	2016	China	SNHG 15	152	Tissue	OS	qRT‐PCR	Median	60	Reported	9
Zhang, M	2016	China	SNHG 1	82	Tissue	OS, RFS	qRT‐PCR	NA	60	K‐M curve	7
Zhang, T	2016	China	SNHG 3	144	Tissue	OS, RFS, DFS	ISH	3	60	Reported	9
Cao, C	2017	China	SNHG 6	160	Tissue	OS, DFS	ISH	NA	60	Reported	9
Lan, T	2017	China	SNHG 12	48	Tissue	OS, RFS	qRT‐PCR	Median	48	K‐M curve	6
Liu, J	2017	China	SNHG 20	96	Tissue	OS	qRT‐PCR	Median	60	K‐M curve	7
Guo, Z	2019	China	SNHG 16	61	Tissue	OS, DFS	ISH	++	60	Reported	9
Lan, T	2019	China	SNHG 10	64	Tissue	OS	qRT‐PCR	Median	72	Reported	8
Lin, Q	2019	China	SNHG 16	88	Tissue	OS	qRT‐PCR	Mean	60	K‐M curve	7
Yang, X	2019	China	SNHG 7	80	Tissue	OS	qRT‐PCR	Median	60	K‐M curve	7
Yao, X	2019	China	SNHG 7	40	Tissue	OS	qRT‐PCR	MedianNA	36	K‐M curve	6
Zhang, PF	2019	China	SNHG 3	70	Tissue	OS	qRT‐PCR	NA	25	K‐M curve	6
Huang, W	2020	China	SNHG 11	57	Tissue	OS	qRT‐PCR	NA	60	K‐M curve	7
Shen, A	2020	China	SNHG 7	100	Tissue	OS	qRT‐PCR	Mean	84	Reported	9
Xie, Y	2020	China	SNHG 7	80	Tissue	OS	qRT‐PCR	NA	60	K‐M curve	7
Zhong, JH	2020	China	SNHG 16	108	Tissue	OS,DFS	qRT‐PCR	Median	60	K‐M curve	7
Chen, W	2021	China	SNHG 15	58	Tissue	OS	qRT‐PCR	2.68	60	K‐M curve	7
Meng, F	2021	China	SNHG 1	115	Tissue	OS	qRT‐PCR	MedianNA	60	Reported	7
Zhang, YX	2021	China	SNHG 22	60	Tissue	OS	qRT‐PCR	NA	150	Reported	7
Zhu, XM	2021	China	SNHG 17	58	Tissue	OS	qRT‐PCR	Median	60	Reported	9
Kunadirek, P	2022	Thailand	SNHG 9	100	Blood	OS	qRT‐PCR	NA	60	Reported	9
Song, W	2022	China	SNHG 18	111	Tissue	OS	qRT‐PCR	Mean	60	Reported	9
Zhang, QJ	2022	China	SNHG 16	158	Tissue	OS	qRT‐PCR	NA	60	Reported	9

Abbreviations: DFS, disease‐free survival; ISH, in situ hybridization; HR, hazard ratio; K‐M curve, Kaplan–Meier curve; NA, not available; NOS, Newcastle‐Ottawa Scale; OS, overall survival; RFS, recurrence‐free survival; qRT‐PCR, quantitative reverse transcription polymerase chain reaction.

### 3.2 The correlation on SNHG expression and HCC prognosis

#### 3.2.1 SNHG expression and OS

**FIGURE 2:**
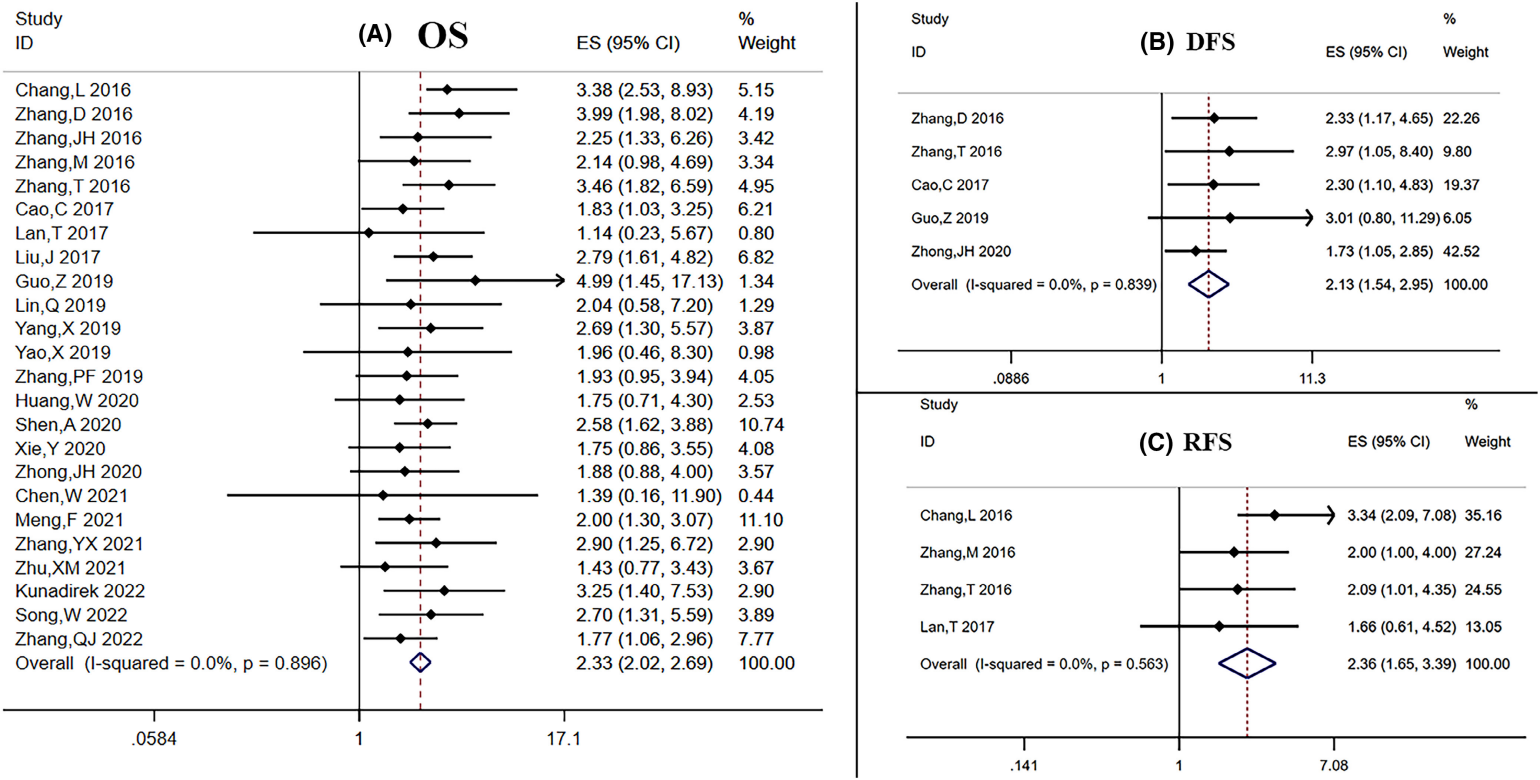
Forest plots of the relationship on SNHG expression and prognosis after deleting “Lan T 2019”: (A) OS, (B) DFS, (C) RFS.

To analyze the relationship of SNHG expression with OS, we selected 25 relevant studies with 2314 participants. The combined results showed a significant association between higher SNHG expression and shorter OS (HR: 2.22, 95% CI: 1.77–2.78, *p*: 0.000, Figure S1). The HR and 95% CI of OS were analyzed using a random‐effects model due to the heterogeneity that existed among the studies (I^2^: 70.5%, *p*: 0.000). Sensitivity analysis shown in Figure S2 revealed that “Lan T 2019” greatly influenced the stability of the results, which may be a possible source of heterogeneity. Then, after removing “Lan T 2019”, the new results showed no heterogeneity (I^2^: 0.00%, *p*: 0.896), indicating that Lan T 2019 was the source of heterogeneity (Figure 2A). As a result, we finally included 24 qualified studies with 2250 patients. A fixed‐effects model determined that the total HR and 95% CI were significant statistically and showed that patients with upregulated SNHGs had a higher risk of having a short OS (HR: 2.33, 95% CI: 2.02–2.69; *p*: 0.000). The results appeared to be trustable, as demonstrated in the sensitivity analysis of Figure S3. Furthermore, in order to carry out a subgroup analysis of OS, all included patients were grouped according to SNHG type, extract method of HR, follow‐up time, and sample size (Table 2). Increased expression of SNHG1, 3, 6, 7, 15, 16, and 20 was substantially connected with poor OS in the SNHG type subgroup, as shown in Figure S4, and other SNHGs were as well. We came to the conclusion that increased expression of SNHGs could result in worse OS both in the group of reported and survival curve when the studies were categorized using the HR extraction approach (reported: HR: 2.44, 95% CI: 2.05–2.90, *p*: 0.000; survival curve: HR: 2.12, 95% CI: 1.64–2.73, *p*: 0.000). In addition, we discovered that HCC patients with a worse prognosis had higher levels of SNHG expression regarding follow‐up time and sample size. The mentioned data supported that SNHGs in HCC patients could serve as prognostic markers for the intervention of OS.

**TABLE 2:** Subgroup analysis of SNHG expression for OS.

Subgroup	No. of studies	No. of patients	HR (95% CI)	*p*	Heterogeneity		
					I^2^ (%)	*p*‐value	Model
OS	24	2250	2.33 (2.02,2.69)	0.000	0	0.890	Fixed
SNHG type							
SNHG 1	2	197	2.03 (1.39, 2.96)	0.000	0	0.882	Fixed
SNHG 3	2	214	2.66 (1.65, 4.29)	0.000	29.7	0.233	Fixed
SNHG 6	2	240	2.42 (1.58, 3.70)	0.000	49.7	0.159	Fixed
SNHG 7	4	300	2.37 (1.72, 3.27)	0.000	0	0.794	Fixed
SNHG 15	2	210	2.13 (1.03, 4.41)	0.042	0	0.680	Fixed
SNHG 16	4	415	2.01 (1.37, 2.95)	0.000	0	0.503	Fixed
SNHG 20	2	240	3.20 (2.08, 4.92)	0.000	0	0.430	Fixed
Others	6	434	2.21 (1.55, 3.13)	0.000	0	0.590	Fixed
Extract method of HR							
Reported	13	1443	2.44 (2.05, 2.90)	0.000	0	0.496	Fixed
K‐M curve	11	807	2.12 (1.64, 2.73)	0.000	0	0.987	Fixed
Follow‐up time							
≥60 months	20	2012	2.32 (2.00, 2.70)	0.000	0	0.862	Fixed
<60 months	4	238	2.42 (1.57, 3.73)	0.000	0	0.494	Fixed
Sample size							
≥80	16	1798	2.41 (2.06, 2.82)	0.000	0	0.837	Fixed
<80	8	452	2.00 (1.41, 2.83)	0.000	0	0.744	Fixed

Abbreviations: 95% CI, confidence interval; HR, hazard ratio.

#### 3.2.2 SNHG expression and DFS or RFS

Figure 2B,C depict the relationship between SNHG expression and DFS, RFS respectively. Five studies^17^, ^20^, ^21^, ^24^, ^32^ consisting of 617 patients demonstrated the utility of SNHGs as predictive biomarkers for DFS in HCC (HR: 2.13, 95% CI: 1.54–2.95; *p*: 0.000). Meanwhile, elevated expression of SNHG in HCC was likewise significantly related to poor RFS, according to four studies^16^, ^19^, ^20^, ^22^ (HR: 2.36, 95% CI: 1.65–3.39, *p*: 0.000).

### 3.3 The SNHG expression and clinicopathological characteristics

In the included papers, we conducted a meta‐analysis to determine whether there was a correlation between the clinicopathological characteristics of HCC and SNHG expression. As presented in Table 3, results showed that elevated SNHG expression was found to be closely related to larger tumor size (OR = 2.42, 95% CI: 1.85–3.15, *p*: 0.000), multiple tumor numbers (OR = 1.66; 95% CI: 1.11–2.49; *p*: 0.013), poor histologic grade (OR = 1.65, 95% CI: 1.31–2.06; *p*: 0.000), advanced tumor stage (OR = 2.71, 95% CI: 2.12–3.47; *p*: 0.000), earlier lymphatic metastasis (OR = 6.28, 95% CI: 2.31–17.10; *p*: 0.000), vein invasion (OR = 2.87, 95% CI: 1.89–4.35, *p*: 0.000), higher alpha‐fetoprotein (AFP) level (OR = 1.61, 95% CI: 1.26–2.06, *p*: 0.000), and portal vein tumor thrombosis (PVTT), (OR = 3.10, 95% CI: 2.19–4.37, *p*: 0.000). Nevertheless, no apparent association was found between overexpression of SNHG and hepatitis B virus (HBV) infection (*p* = 0.886), and cirrhosis (*p* = 0.354).

**TABLE 3:** Meta‐analysis results on the relationship between over‐expressed SNHG and clinicopathological factors.

Clinicopathological parameters	No. of studies	No. of patients	OR (95% CI)	*p*	Heterogeneity			Begg	Egger
					I^2^	*p*‐value	Model		
Tumor size (≥5 cm vs. <5 cm)	17	1706	2.42 (1.85,3.15)	0.000	73.6	0.000	Random	0.048	0.000
Tumor number (multiple vs. solitary)	9	1040	1.66 (1.11,2.49)	0.013	67.3	0.003	Random	0.677	0.284
Differentiation (poor vs. well/moderately)	10	906	1.65 (1.31,2.06)	0.000	14.2	0.312	Fixed	0.107	0.037
Tumor stage	18	1768	2.71 (2.12,3.47)	0.000	71.8	0.000	Random	0.034	0.296
1.TNM (III‐IV vs. I‐II)	10	886	4.07 (2.68,6.20)	0.000	72.8	0.000	Random	–	–
2.BCLC (B + C vs. A)	8	882	2.21 (1.66,2.95)	0.001	47.9	0.062	Random	–	–
3.Edmondson (III‐IV vs. I‐II)	4	512	1.65 (1.24,2.20)	0.000	9.6	0.345	Fixed	–	–
Lymphatic metastasis (yes vs. no)	5	387	6.28 (2.31,17.10)	0.000	75.5	0.003	Random	–	–
Vein metastasis (yes vs. no)	8	666	2.87 (1.89,4.35)	0.000	46.5	0.070	Random	0.386	0.266
AFP	8	799	1.61 (1.26,2.06)	0.000	33.7	0.159	Fixed	0.711	0.730
1.AFP (≥20ug/L vs. <20ug/L)	4	357	1.73 (0.97, 3.11)	0.066	53.7	0.091	Random	–	–
2.AFP (≥400ug/L vs. <400ug/L)	4	442	1.51 (1.11,2.07)	0.026	18.4	0.299	Fixed	–	–
PVTT (yes vs. no)	7	795	3.10 (2.19,4.37)	0.000	45.5	0.088	Random	0.176	0.377
HBV infection (yes vs. no)	9	834	1.03 (0.69,1.53)	0.886	41.6	0.090	Random	0.754	0.537
Liver cirrhosis (yes vs. no)	11	1154	1.10 (0.90,1.36)	0.354	0	0.532	Fixed	0.350	0.147

Abbreviations: 95% CI confidence interval, OR odd ratio.

#### 3.3.1 SNHG and tumor size, number, and differentiation

**FIGURE 3:**
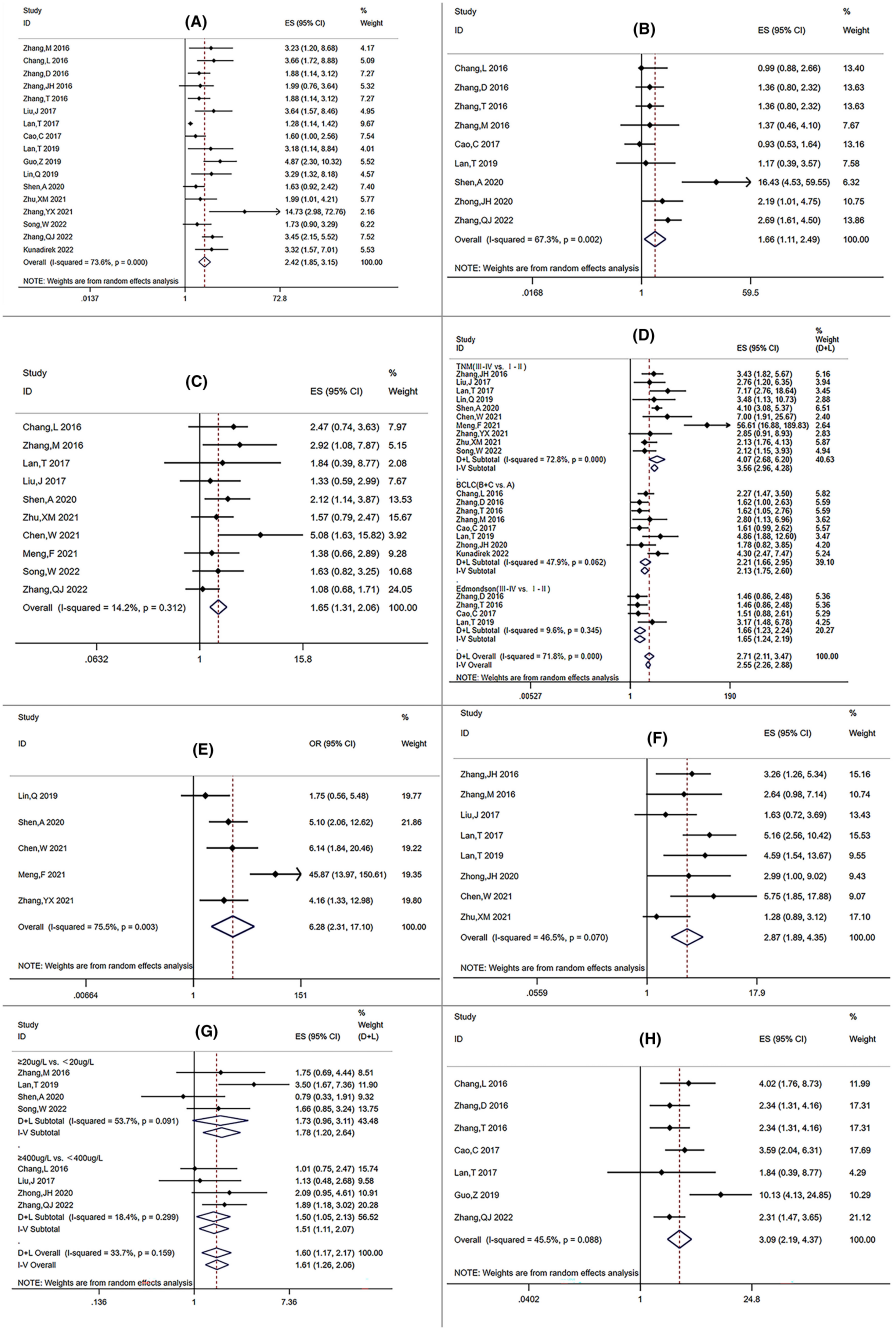
Forest plots on the association between SNHG expression and clinicopathological feactures. (A) tumor size; (B) tumor number; (C) differentiation; (D) tumor stage; (E) lymphatic metastasis; (F) vein Invasion; (G) AFP; (H) portal vein tumor thrombosis (PVTT).

Larger tumor size was found in the group of “high SNHG” in 17 studies (tumor diameter ≥5 cm vs. <5 cm, OR: 2.42, 95% CI: 1.85–3.15, *p*: 0.000; Figure 3A). Given the significant degree of heterogeneity (I^2^: 73.6%, *p*: 0.000), a random‐effects model was used. To examine the association between SNHG and tumor number, nine studies encompassing 1040 patients were analyzed with a random‐effects model (I^2^: 67.3%, *p*: 0.003) (Figure 3B). The findings indicated that patients with multiple tumor numbers were probably accompanied by elevated SNHG levels (OR: 1.66; 95% CI: 1.11–2.49; *p*: 0.013). Meanwhile, adopting a fixed‐effects model with no clear heterogeneity observed (I^2^: 14.2%, *p*: 0.312), 10 studies including 906 patients showed that HCC patients with SNHG overexpression had a higher likelihood of developing a low histological grade (Figure 3C OR: 1.65, 95% CI: 1.31–2.06; *p*: 0.000).

#### 3.3.2 SNHG and tumor stage

We pooled the OR and 95% CI values from 18 research to analyze the relationship on SNHG expression and the HCC stage. A random‐effects model was applied since the heterogeneity between studies was >50% and the p‐value <0.1 (I^2^: 71.8%, P: 0.000). The combined OR and 95% CI had statistical significance (OR: 2.71, 95% CI: 2.12–3.47; P: 0.000), suggesting over‐expressed SNHG was related with the advanced clinical stage (Figure 3D). When subgroup analysis was performed using stage criteria such as TNM, BCLC, and Edmondson stage, the same conclusion was reached (TNM (III‐IV vs. I‐II): OR: 4.07, 95% CI: 2.68–6.20; *p*: 0.000; BCLC (B + C vs. A): OR: 2.21, 95%CI: 1.66–2.95; *p*: 0.001; Edmondson (III–IV vs. I–II): OR: 1.65, 95% CI: 1.24–2.20; *p*: 0.000). Thus, overexpression of SNHG was likely to raise the risk of advanced‐stage HCC.

#### 3.3.3 SNHG and lymphatic metastasis and vein invasion

There were 5 studies involving 387 participants examined the association of SNHG expression and lymphatic metastasis. The total OR and 95% CI were calculated using a random‐effects model due to the heterogeneity among studies (I^2^: 75.5%, *p*: 0.003; Figure 3E). According to the findings, early lymph node metastasis was linked to upregulated SNHG expression in HCC patients (OR: 6.28, 95% CI: 2.31–17.10; *p*: 0.000). Meanwhile, patients with high SNHG expression had a higher risk of vein invasion than those with low expression, according to the pooled results from eight high‐quality articles (Figure 3F, OR: 2.87, 95% CI: 1.89–4.35, *p*: 0.000) by a random‐effects model (I^2^: 46.5%, *p*: 0.070).

#### 3.3.4 SNHG with AFP and PVTT

The relationship on SNHG expression and the AFP value of patients was explored in 8 studies. No notable heterogeneity among the research was found, thus the fixed‐effects model with an OR of 1.61 (95% CI: 1.26–2.06, *p*: 0.000) (Figure 3G), showed that increased levels of SNHGs tended to be accompanied by higher AFP value in HCC. Furthermore, four studies of them had an AFP cutoff value of 20ug/L and the others were 400ug/L. AFP >400ug/L was substantially related to high expression of SNHG, while AFP >20ug/L exhibited no noticeable difference, according to the subgroup analysis carried out depending on the cutoff values (AFP (≥20ug/L vs. 20ug/L): OR: 1.73, 95% CI: 0.97–3.11, *p*: 0.066; AFP (≥400ug/L vs. <400ug/L): OR: 1.51, 95% CI: 1.11–2.07, *p*: 0.026). Then, to see if SNHG expression was related to combined PVTT in HCC patients, we analyzed seven studies involving 795 patients (Figure 3H). The random‐effects model we chose revealed that PVTT was associated with increased SNHG expression in HCC patients due to the high heterogeneity among studies (I^2^: 45.5%, *p*: 0.088; OR: 3.10, 95% CI: 2.19–4.37, *p*: 0.000). Besides, the relationship between SNHG expression and other clinical characteristics was also explored such as age (OR: 1.05, 95% CI: 0.88–1.26, *p*: 0.605), gender (OR: 1.05, 95% CI: 0.85–1.28, *p*: 0.665), HBV infection (OR: 1.03, 95% CI: 0.69–1.53, *p*: 0.886) and cirrhosis (OR: 1.10, 95% CI: 0.90–1.36, *p*: 0.354). However, no significant associations were found.

### 3.4 Publication bias

**FIGURE 4:**
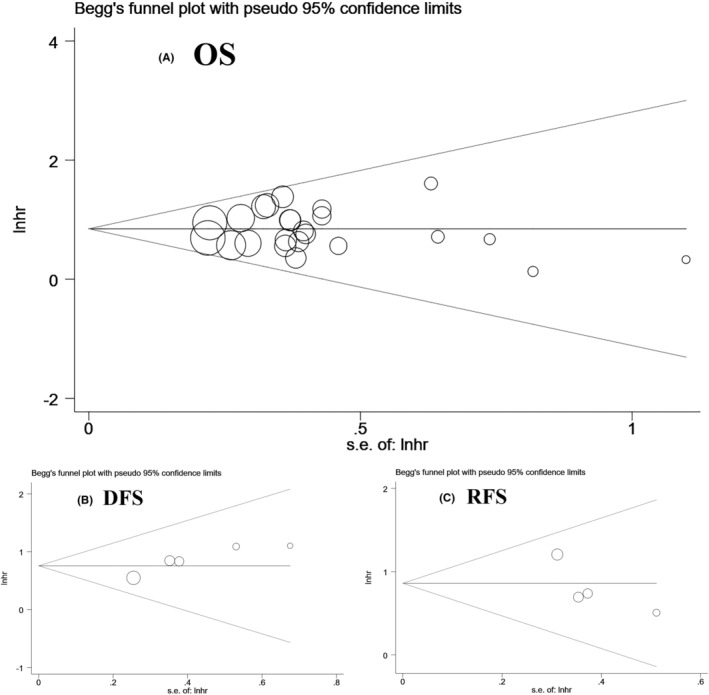
Begg's funnel plot of publication bias for OS, DFS, and RFS. Each circle represents an independent study.

The funnel plot, Begg's test, and Egger's test were used to examine potential publication bias. The meta‐analysis for OS, DFS, and RFS did not exhibit any publication bias, according to Begg's test and Egger's test (OS: *p*: 0.862 and *p*: 0.850, respectively; DFS: *p*: 0.221 and *p*: 0.021, respectively; RFS: *p*: 0.308 and *p*: 0.207, respectively). Begg's funnel plot in Figure 4 was generally symmetrical. In addition, we assessed publication bias in terms of clinicopathological characteristics, including age, gender, histological grade, vein invasion, cirrhosis, tumor number, tumor size, HBV infection, PVTT, tumor stage, and AFP (Table 3).

## 4 DISCUSSION

LncRNAs are RNA molecules with a length more than 200 nucleotides that are incapable of being translated into proteins. Recent research has shown that lncRNAs regulate gene expression, which have a profound impact on various physiological and pathological processes. In particular, they can function as oncogenes or anti‐oncogenes that directly or indirectly control signaling pathways associated with tumors and influence tumor progression.^41^ Due to the elevated activation of carcinogenic lncRNAs in various cancers in adjacent tissues, concern over lncRNAs as diagnostic or prognostic markers is growing.^42^ Similarly, The SNHGs may also be promising indicators for the detection and prognosis of cancer according to many research.

It has been extensively studied how SNHGs contribute to the growth of HCC. SNHGs can influence the biologically malignant behavior of tumors through acting as endogenous RNAs (ceRNAs) to adsorb numerous miRNAs, directly binding to and upregulating mRNA, interacting with transcription factors to activate transcription, and activating the signaling pathways such as Wnt/β‐catenin that are primarily part in tumor growth. They significantly affected the outcome of HCC patients by promoting growth and metastasis or interfering with apoptosis and autophagy in HCC.^12^ Zhang et al^19^ stated SNHG1 drastically improved the proliferation and migration of HCC by suppressing the activity of p53 and its target genes, which also inhibited the apoptosis process. According to the study by Xie et al,^32^ SNHG7, which was highly expressed in HCC, might compete with miR‐9‐5p as a ceRNA. As a result, it could upregulate the activity of the CNNM1, which encouraged the growth of HCC in turn. The majority of SNHGs functioned as ceRNAs to promote malignancy in HCC. In addition, SNHGs also played important roles in HCC via additional mechanisms. For example, in HCC, SNHG3,^29^ SNHG7,^28^ and SNHG20^23^ promoted epithelial‐to‐mesenchymal transition (EMT), and SNHG16^40^ significantly activated the ECM‐receptor interaction pathway, and so on. To sum up, mechanisms of SNHGs varied in HCC. Therefore, we carried out a further analysis to determine if SNHGs were valuable in the prognosis of HCC.

In our analysis to summarize the relationship on prognosis and clinicopathological parameters with SNHG in HCC, a total of 25 eligible papers satisfying inclusion criteria were taken into account. Patients with high SNHG expression had a higher chance of undergoing shorter OS, DFS, and RFS compared to those with low expression. Additionally, the association between SNHG and OS under various situations was further investigated with subgroup analysis. All pooled results showed that SNHG overexpression was related to worse OS of HCC in subgroups of SNHG type, extract method, follow‐up time, and sample size.

The correlation on SNHG expression and various clinicopathological features in HCC was also explored at the same time. Patients who had high levels of SNHGs expression were inclined to have larger tumor size, multiple tumors, worse histologic grades, more advanced tumor stage, positive lymphatic metastasis, vein invasion, PVTT, and AFP values >400ug/L. In contrast, SNHG expression had no bearing on a range of other clinical characteristics, including age, gender, HBV infection, and cirrhosis. Taking into account all of these findings, lncRNA SNHGs played a part in the emergence of HCC and had the potential to grow into a biomarker for the prognosis.

Our findings were consistent with a meta‐analysis^13^ on SNHG and HCC prognosis in 2017, which covered only 5 papers at that time. In recent years, more studies on this subject have been published. Then, we reanalyzed the association between SNHG and clinical features along with the prognosis of HCC, which appeared more convincing and detailed.

However, we should acknowledge some limitations of the study. Firstly, the results couldn't be generalized to other nations because all but one of the research included in it were from China, which could have caused publication bias. Secondly, there was a chance of errors because the HRs and 95% CIs were partially extracted indirectly from the survival curves. Thirdly, the cutoff values of SNHG varied between research, which could have an impact on the relevant outcomes. Lastly, only a portion of SNHGs was involved in our analysis due to insufficient research and sample size.

## 5 CONCLUSION

To summarize, we conclude from this meta‐analysis that upregulation of SNHG relates to malignant clinicopathological features of HCC and has potential as a prognostic biomarker. Future large‐scale, multicenter studies will be necessary to confirm our findings.

## AUTHOR CONTRIBUTIONS

**Meng Huang:** Data curation (lead); methodology (lead); software (lead); writing – original draft (lead). **Lihua Yang:** Conceptualization (lead); supervision (lead); writing – review and editing (lead). **Zhiwen Zhao:** Formal analysis (lead); investigation (lead); resources (equal); validation (lead); visualization (lead).

## FUNDING INFORMATION

NA.

## CONFLICT OF INTEREST STATEMENT

The authors declare no conflict of interest.

## ETHICS STATEMENT

This article does not contain any studies with human participants or animals performed by any author.

## Supporting information


Data S1.

## Data Availability

All data generated or analyzed during this study are included in this published article and its supporting information.
