# Supplementary material for: Long noncoding RNA small nucleolar RNA host genes as prognostic molecular biomarkers in hepatocellular carcinoma: A meta‐analysis
Source: Cancer Med. 2024 Apr 17;13(8):e7200. doi: 10.1002/cam4.7200 (PMC11024508; doi:10.1002/cam4.7200)
Supplement: Supplementary file 1 — Data S1. [file CAM4-13-e7200-s001.docx]

**Supporting information**

**
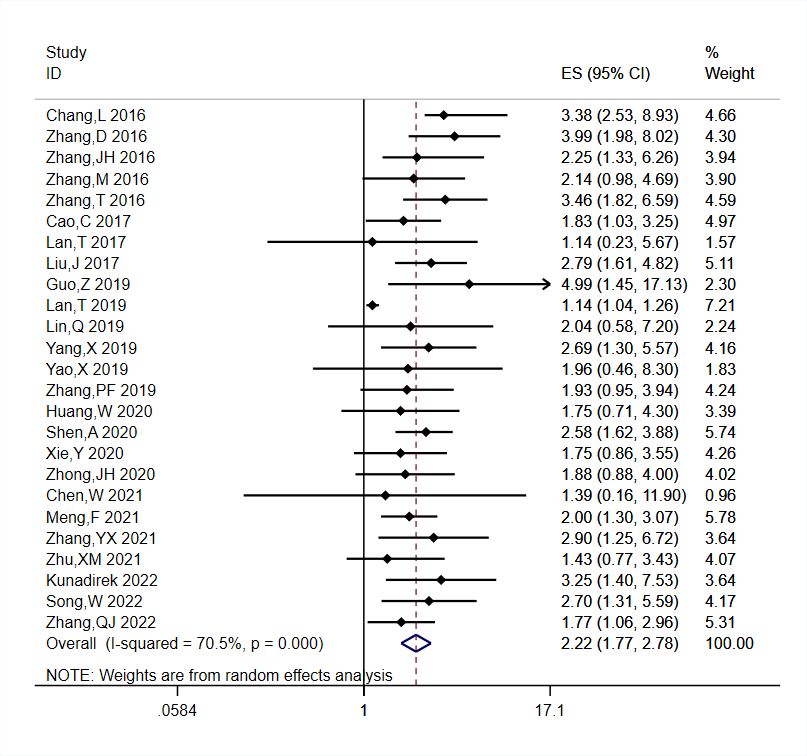
**

**Supporting Figure 1.** Forest plot for the relationship between SNHG expression and OS before deleting “Lan T 2019”.

**
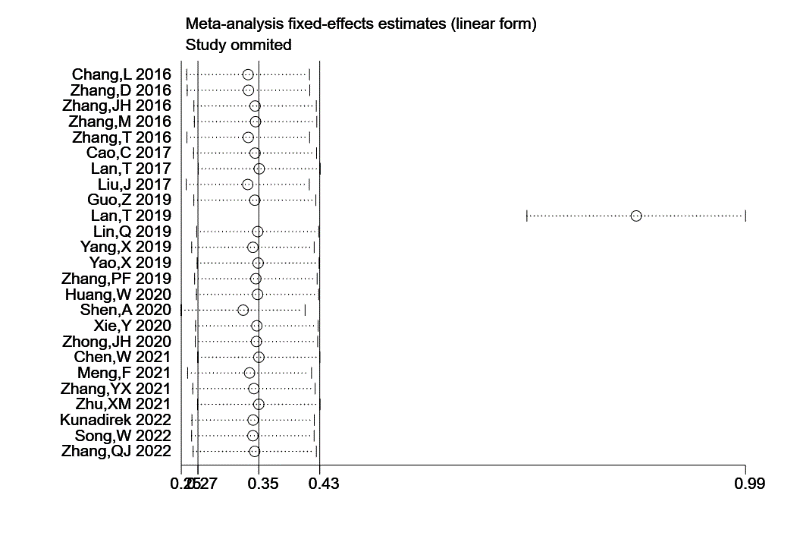
**

**Supporting Figure 2.** Sensitivity analysis for meta-analysis of SNHG and OS before deleting “Lan T 2019”.


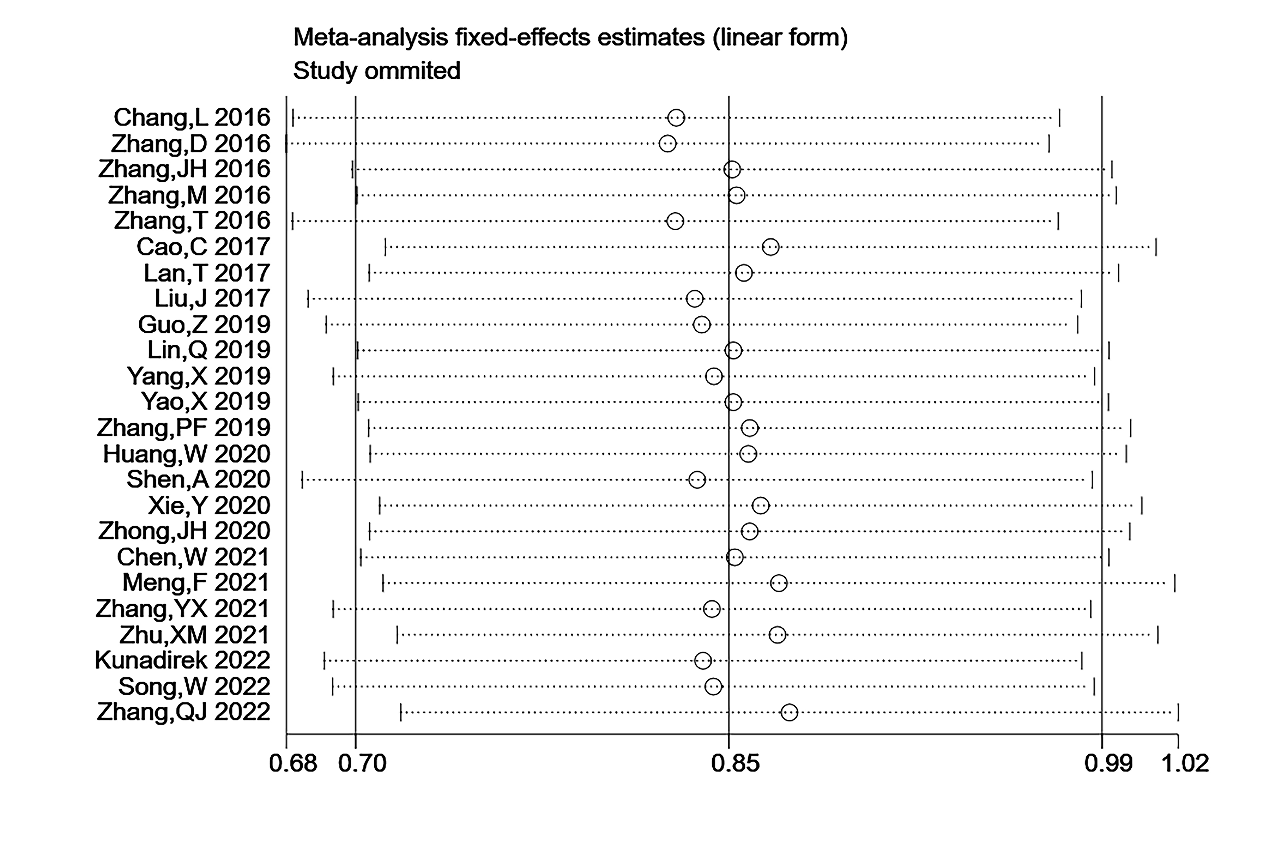


**Supporting Figure 3.** Sensitivity analysis of meta-analysis of SNHG and OS after deleting “Lan T 2019”.

**
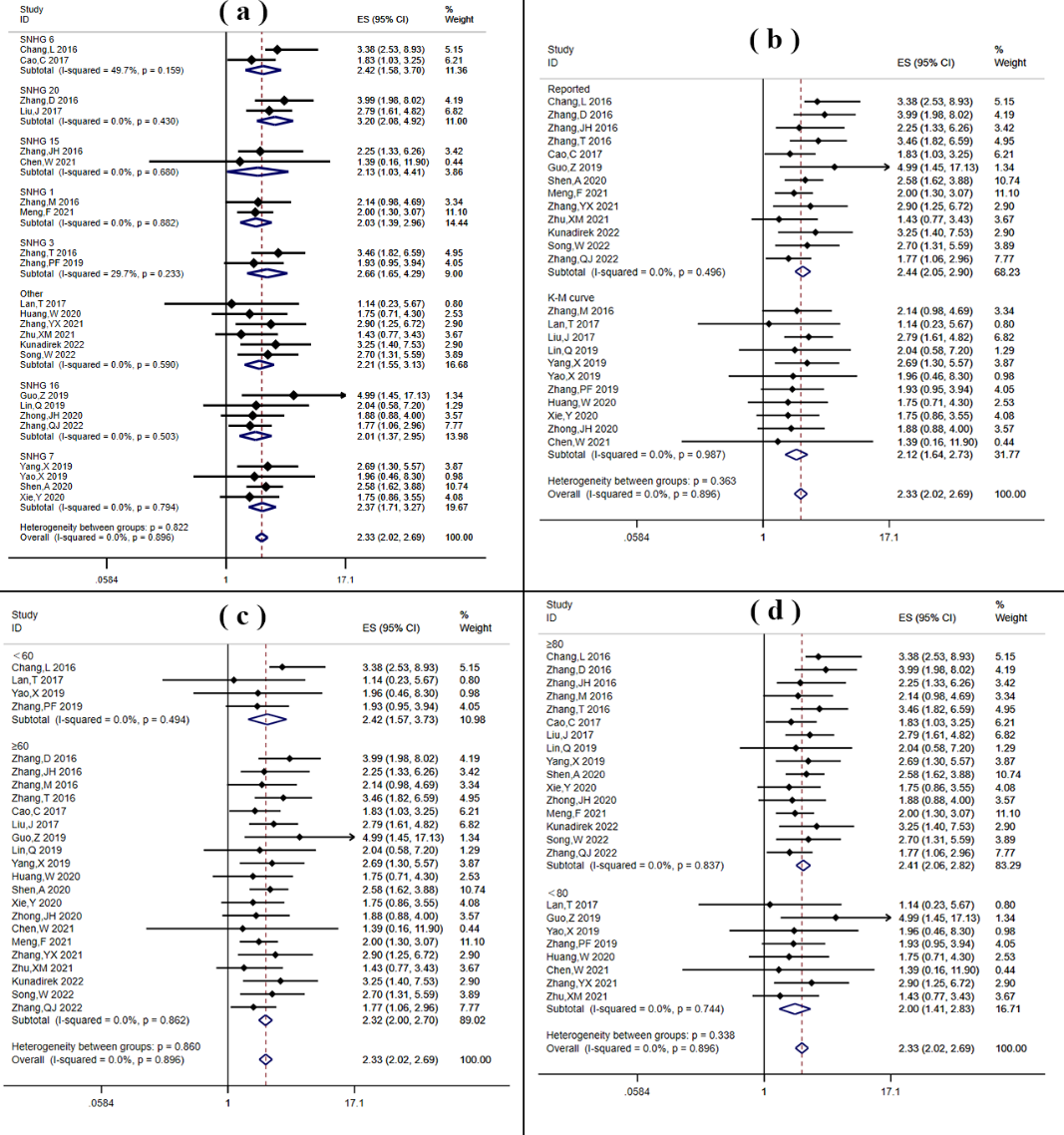
**

**Supporting Figure 4.** Forest plots of hazard ratios (HRs) for the relationship between SNHG expression and OS: (a) SNHG type; (b)extract method of HR; (c) follow-up time; (d) sample size.

**Supporting Table 1.** Full search strategy employed for each database (up to and including Feb 10th, 2024).

| **DATABASE** | **SEARCH STRATEGY** |
| --- | --- |
| **PubMed** | (SNHG1 OR SNHG2 OR SNHG3 OR SNHG4 OR SNHG5 OR SNHG6 OR SNHG7 OR SNHG8 OR SNHG9 OR SNHG10 OR SNHG11 OR SNHG12 OR SNHG13 OR SNHG14 OR SNHG15 OR SNHG16 OR SNHG17 OR SNHG18 OR SNHG19 OR SNHG20 OR SNHG21 OR SNHG22 ) AND ((Hepatocellular Carcinoma) OR (Liver Cancer) OR (Liver Cell Carcinoma) OR (Hepatoma)) AND (prognosis OR prognostic OR outcome) |
| **Embase** | #1 = snhg1 OR snhg2 OR snhg3 OR snhg4 OR snhg5 OR snhg6 OR snhg7 OR snhg8 OR snhg9 OR snhg10 OR snhg11 OR snhg12 OR snhg13 OR snhg14 OR snhg15 OR snhg16 OR snhg17 OR snhg18 OR snhg19 OR snhg20 OR snhg21 OR snhg22  #2 = 'hepatocellular carcinoma'/exp OR 'hepatocellular carcinoma' OR (hepatocellular AND ('carcinoma'/exp OR carcinoma)) OR 'liver cancer'/exp OR 'liver cancer' OR (('liver'/exp OR liver) AND ('cancer'/exp OR cancer)) OR 'liver cell carcinoma'/exp OR 'liver cell carcinoma' OR (('liver'/exp OR liver) AND ('cell'/exp OR cell) AND ('carcinoma'/exp OR carcinoma)) OR 'hepatoma'/exp OR hepatoma  #3 = 'prognosis'/exp OR prognosis OR prognostic OR outcome  #4 = #1 AND #2 AND #3 |
| **Web of Science** | #1 = (((ALL=(Hepatocellular Carcinoma)) OR ALL=(Liver Cancer)) OR ALL=(Liver Cell Carcinoma)) OR ALL=(Hepatoma)  #2 = (((((((((((((((((((((ALL=(SNHG1)) OR ALL=(SNHG2)) OR ALL=(SNHG3)) OR ALL=(SNHG4)) OR ALL=(SNHG5)) OR ALL=(SNHG6)) OR ALL=(SNHG7)) OR ALL=(SNHG8)) OR ALL=(SNHG9)) OR ALL=(SNHG10)) OR ALL=(SNHG11)) OR ALL=(SNHG12)) OR ALL=(SNHG13)) OR ALL=(SNHG14)) OR ALL=(SNHG15)) OR ALL=(SNHG16)) OR ALL=(SNHG17)) OR ALL=(SNHG18)) OR ALL=(SNHG19)) OR ALL=(SNHG20)) OR ALL=(SNHG21)) OR ALL=(SNHG22)  #3 = ((ALL=(prognosis)) OR ALL=(prognostic)) OR ALL=(outcome)  #4 = #1 AND #2 AND #3 |
| **Cochrane Library** | ("SNHG1" OR "SNHG2" OR "SNHG3" OR "SNHG4" OR "SNHG5" OR "SNHG6" OR "SNHG7" OR "SNHG8" OR "SNHG9" OR "SNHG10" OR "SNHG11" OR "SNHG12" OR "SNHG13" OR "SNHG14" OR "SNHG15" OR "SNHG16" OR "SNHG17" OR "SNHG18" OR "SNHG19" OR "SNHG20" OR "SNHG21" OR "SNHG22") AND ("Hepatocellular Carcinoma" OR "liver cancer" OR "Liver Cell Carcinoma" OR "Hepatoma") AND ("prognosis" OR "prognostic" OR "outcome"):ti, ab, kw |

**Supporting Table 2.** NOS quality scores of included studies.

| Study | Selection | | | | Comparability | Outcome | | | Sco  re |
| --- | --- | --- | --- | --- | --- | --- | --- | --- | --- |
|  | Representativeness of the exposed cohort | Selection of the nonexposed cohort | Ascertainment of exposure | Demonstration that outcome of interest was not present at the start of study | Comparability of cohorts on the basis of the design or analysis | Assessment of outcome | Was follow-up long enough for outcomes to occur | Adequacy of follow up of cohorts |  |
| Chang,L 2016 | ★ | ★ | ★ | ★ | ★★ | ★ |  | ★ | 8 |
| Zhang,D 2016 | ★ | ★ | ★ | ★ | ★★ | ★ | ★ | ★ | 9 |
| Zhang,JH 2016 | ★ | ★ | ★ | ★ | ★★ | ★ | ★ | ★ | 9 |
| Zhang,M 2016 | ★ | ★ | ★ | ★ |  | ★ | ★ | ★ | 7 |
| Zhang,T 2016 | ★ | ★ | ★ | ★ | ★★ | ★ | ★ | ★ | 9 |
| Cao,C 2017 | ★ | ★ | ★ | ★ | ★★ | ★ | ★ | ★ | 9 |
| Lan,T 2017 | ★ | ★ | ★ | ★ |  | ★ |  | ★ | 6 |
| Liu,J 2017 | ★ | ★ | ★ | ★ |  | ★ | ★ | ★ | 7 |
| Guo,Z 2019 | ★ | ★ | ★ | ★ | ★★ | ★ | ★ | ★ | 9 |
| Lan,T 2019 | ★ | ★ | ★ | ★ | ★ | ★ | ★ | ★ | 8 |
| Lin,Q 2019 | ★ | ★ | ★ | ★ |  | ★ | ★ | ★ | 7 |
| Yang,X 2019 | ★ | ★ | ★ | ★ |  | ★ | ★ | ★ | 7 |
| Yao,X 2019 | ★ | ★ | ★ | ★ |  | ★ |  | ★ | 6 |
| Zhang,PF 2019 | ★ | ★ | ★ | ★ |  | ★ |  | ★ | 6 |
| Huang,W 2020 | ★ | ★ | ★ | ★ |  | ★ | ★ | ★ | 7 |
| Shen,A 2020 | ★ | ★ | ★ | ★ | ★★ | ★ | ★ | ★ | 9 |
| Xie,Y 2020 | ★ | ★ | ★ | ★ |  | ★ | ★ | ★ | 7 |
| Zhong,JH 2020 | ★ | ★ | ★ | ★ |  | ★ | ★ | ★ | 7 |
| Chen,W 2021 | ★ | ★ | ★ | ★ |  | ★ | ★ | ★ | 7 |
| Meng,F 2021 | ★ | ★ | ★ | ★ |  | ★ | ★ | ★ | 7 |
| Zhang,YX 2021 | ★ | ★ | ★ | ★ |  | ★ | ★ | ★ | 7 |
| Zhu,XM 2021 | ★ | ★ | ★ | ★ | ★★ | ★ | ★ | ★ | 9 |
| Kunadirek 2022 | ★ | ★ | ★ | ★ | ★★ | ★ | ★ | ★ | 9 |
| Song,W 2022 | ★ | ★ | ★ | ★ | ★★ | ★ | ★ | ★ | 9 |
| Zhang,QJ 2022 | ★ | ★ | ★ | ★ | ★★ | ★ | ★ | ★ | 9 |
